# Genome-Wide Identification of the *NFYA* Family and Its Expression in Response to Abiotic Stress in *Taxodium* Hybrid ‘Zhongshanshan’

**DOI:** 10.3390/life16081358

**Published:** 2026-08-19

**Authors:** Minyue Cai, Tingting Chen, Zijing Guo, Wanwen Yu, Yunlong Yin, Chaoguang Yu, Yan Lu

**Affiliations:** 1 Jiangsu Provincial Key Laboratory for Plant Taxonomy, Resource Conservation and Utilization, Institute of Botany, Jiangsu Province and Chinese Academy of Sciences (Nanjing Botanical Garden Mem. Sun Yat-Sen), Nanjing 210014, China; caimao1113@126.com (M.C.); tingtingchen622@163.com (T.C.); yinyl066@sina.com (Y.Y.); yuchaoguang168@cnbg.net (C.Y.); 2 College of Forestry, Henan Agricultural University, Zhengzhou 450046, China; 3 State Key Laboratory of Tree Genetics and Breeding, Nanjing 210037, China; 18506335567@163.com (Z.G.); youeryuww@163.com (W.Y.); 4 State Key Laboratory for Development and Utilization of Forest Food Resources, Co-Innovation Center for Sustainable Forestry in Southern China, Nanjing Forestry University, Nanjing 210037, China

**Keywords:** *Taxodium*, transcription factor, NFYA, environmental stress, flooding

## Abstract

*Nuclear Factor Y*, *subunit A* (*NFYA*) constitutes a family of transcription factors that play critical roles in plant growth, development and abiotic stress responses. *Taxodium* hybrid ‘Zhongshanshan’ (*T. mucronatum* × *T. distichum*) is a fast-growing tree species with high industrial value and remarkable flooding tolerance. However, the systematic characteristics and abiotic stress response patterns of the *ThNFYA* gene family remain unclear. In this study, a total of 11 *ThNFYA* genes were identified. The encoded proteins ranged from 67 to 372 amino acids in length, with predicted molecular weights between 16.84 and 40.12 kDa. Phylogenetic analysis classified plant NFYAs into four clades, with all ThNFYAs falling into clades I and IV. Expression profiling revealed tissue-specific patterns, with six members showing the highest transcript levels in the cambium. Multiple cis-acting elements associated with stress and hormone responses were detected in the promoter regions of *ThNFYAs*. Most *ThNFYAs* were differentially regulated under salt, drought, and flooding stresses. Notably, most clade IV members (*ThNFYA3*, *ThNFYA4*, and *ThNFYA6*-*ThNFYA8*) were downregulated in the wood under partial submergence. This indicates their potential role in modifying wood properties in response to flooding. Co-expression network analysis identified *ThNFYA1* and *ThNFYA8* as central hub genes in leaves under partial submergence. Overall, these results suggest that the *ThNFYA* family may serve as candidate regulators of development and stress adaptation in *T*. hybrid ‘Zhongshanshan’. This study provides valuable insights for further functional verification of *ThNFYAs* and lays a foundation for marker-assisted breeding of stress-tolerant varieties.

## 1. Introduction

The Nuclear Factor Y (NFY) family is widely present in eukaryotes and typically functions as a heterotrimeric complex consisting of NFYA, NFYB, and NFYC subunits. This complex can specifically recognize and bind to the CCAAT element in gene promoter regions. The CCAAT motif is widely distributed in a large proportion of genes, enabling the NFY complex to serve as a core regulator of transcriptional regulation for diverse downstream target genes [1]. Among these subunits, NFYA is particularly critical, acting as the essential subunit for heterotrimer assembly and subsequent DNA binding activity [2]. While mammals and yeast possess only one or two *NFYA* genes, this family has undergone significant expansion in plants [3]. For instance, 10, 10 and 16 NFYA members have been identified in *Arabidopsis thaliana*, maize (*Zea mays*) and rice (*Oryza sativa*), respectively [4,5,6], suggesting the evolution of more complex and specialized regulatory functions [7]. An evolutionary analysis across 320 plant species showed that *NFY* genes originated from charophytes, and that angiosperms expanded this family predominantly through whole-genome duplication and tandem duplication [8]. In contrast, bryophytes, pteridophytes, and gymnosperms relied mainly on dispersed duplication to expand their *NFY* gene repertoires [8].

In plants, NFYAs have been well documented as important regulators of diverse developmental processes, including embryogenesis, root development and flowering time [3]. For instance, tissue-specific expression patterns of *NFYA* members in *Arabidopsis* imply their involvement in various developmental processes [9]. Overexpression of *AtNFYA2* and *AtNFYA10* promotes leaf growth via auxin signaling [10], and *AtNFYA3* and *AtNFYA8* regulate cell differentiation and embryo formation [11]. In poplars, the mRNA levels of *NFYAs* are tightly correlated with the cambial activity, implying their potential involvement in secondary growth [12,13,14]. In gymnosperms, a multi-omics analysis has identified *PkNFYA7a* as a key transcriptional activator of glycerolipid biosynthesis during seed development in *Pinus koraiensis* [15].

NFYAs also function as critical regulators in plant adaptive responses to abiotic stresses, such as salinity, drought and cold [16]. They confer tolerance largely by transcriptionally activating downstream genes involved in osmotic adjustment, reactive oxygen species scavenging, membrane protection and abscisic acid (ABA) signaling [17,18]. For instance, overexpression of *ZmNFYA1* significantly enhances drought tolerance in maize (*Zea mays*) by upregulating the expression of *α-amylase* (*ZmAMY*), *peroxidase 64* (*ZmPOD64*), and *lipoxygenase 5* (*ZmLOX5*) [19,20]. In *Arabidopsis*, *AtNFYA1* enforces post-germination growth arrest under salt stress by activating core ABA signaling components *ABI3* and *ABI5* [21]. In poplars, a miR169y-*PtrNFYA6*-*PtrNCED3a/3b/6* cascade enhances drought resilience via modulating ABA production [22]. Although flooding represents a major environmental constraint affecting pant growth and unelucidated, the roles of *NFYAs* in flooding adaptation have remained largely unexplored. In wheat (*Triticum aestivum*), most *TaNF*-YAs are differentially expressed under waterlogging [23]. To date, only one study has validated the functional role of *NFYAs* in flooding tolerance, demonstrating that overexpression of *AtNFYA2* and *AtNFYA7* markedly reduces submergence tolerance in *A. thaliana* [24]. Notably, the functions of *NFYAs* in trees under flooding stress remain largely unknown.

As ancient woody gymnosperms, *Taxodium* species are renowned for their exceptional adaptation to flooded environments. *T*. hybrid ‘Zhongshanshan’ (Figure S1), as a hybrid derived from *T. mucronatum* and *T. distichum*, exhibits notable stress resilience, rapid growth abilities and high ornamental value [25]. This hybrid also possesses superior economic and industrial value, serving as a source of essential oil and wood products. More importantly, it is extremely flooding-tolerant. In the Three Gorges Reservoir area, over 90% of *T*. hybrid ‘Zhongshanshan’ individuals can survive continuous submergence for up to 271 days [26,27]. The chromosome-level genome assembly of baldcypress revealed that this species employs ERF-VII transcriptional activation and antioxidant pathways to withstand flooding, representing an adaptive strategy distinct from that of rice [28]. However, genome-wide analysis of *NFYAs*, as key master regulators of stress-responsive transcription, has not been conducted in any *Taxodium* species. The roles of *NFYAs* in flooding stress have rarely been investigated. Here, we systematically identified *NFYA* genes in *T*. hybrid ‘Zhongshanshan’, characterized their structural features, and analyzed their expression profiles under saline, dehydration, and flooding. This study will not only expand our knowledge of *NFYA* in woody angiosperms but also lay a foundation for further genetic improvement of stress tolerance in trees.

## 2. Materials and Methods

### 2.1. Identification of NFYAs in T. Hybrid ‘Zhongshanshan’

The identification of the *NFYA* gene family in *T*. hybrid ‘Zhongshanshan’ was performed according to previous established methods [29,30] with minor modifications. Briefly, the protein sequences of AtNFYA members from *A. thaliana* (https://www.Arabidopsis.org/, accessed on 19 July 2026) were used as queries to perform BLASTP searches against the reference genome of *T. distichum* [28]. A hidden Markov model (HMM) was constructed based on the conserved CBF_NFYA domain (Pfam: PF02045), and was subsequently used to search the reference genome using HMMER software (v3.0). After integrating results from BLASTP and HMMER analyses, the overlapping genes were retained as putative *ThNFYAs*. To ensure domain integrity, these candidates were further validated using the NCBI Conserved Domain Database and Pfam database. The basic physicochemical properties of ThNFYAs, such as amino acid length and molecular weight, were assessed using ExPASy (https://web.expasy.org/protparam/, accessed on 19 July 2026). WoLF PSORT (https://wolfpsort.hgc.jp/, accessed on 19 July 2026) was used to predict the subcellular localization of each ThNFYA. TBtools [31] was employed to visualize the chromosomal locations of the *ThNFYAs*, based on the gff file of the *T. distichum* genome.

### 2.2. Investigating the Phylogeny, Gene Architecture, and Conserved Domains of ThNFYAs

For phylogenetic analysis, protein sequences of NFYA members from *Arabidopsis thaliana* (At), *Pinus tabuliformis* (Pita), *Populus trichocarpa* (Ptr) and *Z*. *mays* (Zm) were acquired from the Phytozome database (https://phytozome-next.jgi.doe.gov/, accessed on 19 July 2026). MEGA11 was employed to build the phylogenetic tree using the neighbor-joining (NJ) criterion with 1000 bootstrap replicates. The obtained tree was subsequently displayed on iTOL (https://itol.embl.de/, accessed on 19 July 2026) [32].

The exon-intron organization of the *ThNFYAs* was determined based on their coding sequences and genomic sequences. The resulting structure features were visualized using TBtools.

The conserved motifs identification for the *ThNFYAs* were performed on the MEME platform (https://meme-suite.org/meme/tools/meme, accessed on 19 July 2026) under a maximum-motif setting of 10. The results were visualized using TBtools.

### 2.3. Cis-Regulatory Elements Identification in Promoter Regions

The 2000 bp sequences located upstream of *ThNFYAs* were retrieved from *T. distichum* genome. The cis-elements within these promoter sequence were identified using PlantCARE (http://bioinformatics.psb.ugent.be/webtools/plantcare/html/, accessed on 19 July 2026).

### 2.4. Plant Cultivation and Treatments

One-year-old cuttings of *T*. hybrid ‘Zhongshanshan’ were rooted and planted in plastic pots containing soil. The physicochemical properties of the cultivation soil have been described previously [26]. After one year, to evaluate the expression abundance of *ThNFYAs* in different tissues, samples were collected from the cambium, bark, wood, root and leaf tissues.

For salt treatment, 30 saplings with similar height (ca. 30 cm) were transferred to a hydroponics system supplemented with 200 mM NaCl. This concentration was validated as an effective salt-stress condition for *T*. hybrid ‘Zhongshanshan’ in our group’s previous study [33]. The salt stress was lasted for 14 days. The leaves and roots, as the primary tissues responsible for ion absorption and oxidative damage response, were harvested at 0 (serving as control), 1, 3, 7 and 14 days after treatment initiation. These time-points were designed to capture both the early osmotic phase (1–3 days) and the later toxic phase (7–14 days) of salt stress responses.

For drought treatment, 18 saplings with similar height (ca. 30 cm) were exposed to drought via withholding water. Based on preliminary observations, no visible leaf wilting was observed after 10 days of water withholding, whereas clear wilting symptoms appeared after 20 days. Correspondingly, the leaves and roots were harvested at 0 (serving as control), 10 (early adaptive stage) and 20 (severe stress stage) days after the initiation of water withholding. These two tissues were selected due to their critical roles in water sensing and drought symptom formation.

At each sampling time point, six plants were harvested and divided into three biological replicates (two plants per replicate). The collected tissues were rapidly frozen in liquid nitrogen, and then milled to fine powder for the subsequent analysis.

### 2.5. RNA Isolation and Quantitative Real-Time PCR (qRT-PCR) Detection

The FastPure Plant Total RNA Isolation Kit (Vazyme, Nanjing, China) was employed for total RNA extraction. qRT-PCR was performed on a StepOnePlus Real-Time PCR System (Thermo Scientific, Waltham, MA, USA, USA) with ChamQ Universal SYBR qPCR Master Mix (Vazyme) as suggested previously [34]. Gene-specific primers were present in Table S1. *ThActin* was used as an internal control (Table S1). Each qRT-PCR reaction was conducted with three technical replicates.

Statistical evaluation was conducted via one-way ANOVA coupled with Duncan’s multiple range test, implemented in Statgraphics (STN, St Louis, MO, USA) as suggested previously [35]. The assumptions of normality were confirmed before statistical comparison. Exact *p*-values were calculated for all pairwise comparisons, with statistical significance defined as *p* < 0.05.

### 2.6. Expression Levels of ThNFYAs Based on Public Transcriptomic Data

Transcriptomic data for *T*. hybrid ‘Zhongshanshan’ leaves and wood grown under partial submergence were acquired from Genome Sequence Archive (https://ngdc.cncb.ac.cn/gsa/, accessed on 19 July 2026) under project IDs of CRA015226 and CRA019997, respectively [25]. The establishment of hypoxic conditions under partial submergence has been validated by our previous physiological studies [26,27]. Significantly increased activities of alcohol dehydrogenase (ADH) and lactate dehydrogenase (LDH), and elevated lactate concentrations have been observed in the leaves and roots under partial submergence [26,27]. The clean reads were aligned against the *T. distichum* reference genome using Hisat2 software (v2.0.5) [36]. Gene expression levels were estimated and normalized as FPKM (Fragment Per Kilobase of exon per Million fragments mapped). Genes with log_2_(foldchange) more than 1 (or less than −1) and adjusted *p*-value (false discovery rate, FDR) less than 0.05 were defined as significant differentially expressed.

### 2.7. Co-Expression Network Analysis of ThNFYAs and Differentially Expressed Transcription Factors Under Partial Submergence

To explore potential regulatory relationships between *ThNFYAs* and transcription factors (TFs) under flooding stress, a co-expression network analysis was performed using the leaf transcriptomic data under partial submergence as described above. Pearson correlation coefficients (r) and corresponding *p*-values were calculated between the expression patterns of *ThNFYAs* and the differentially expressed TFs using the psych package in R. A significant correlation coefficient was defined as |r| > 0.7 and *p*-value < 0.05. The co-expression network was visualized using Cytoscape (v3.10.1).

## 3. Results

### 3.1. Identification of NFYA Family Genes in T. Hybrid ‘Zhongshanshan’

**Figure 1:**
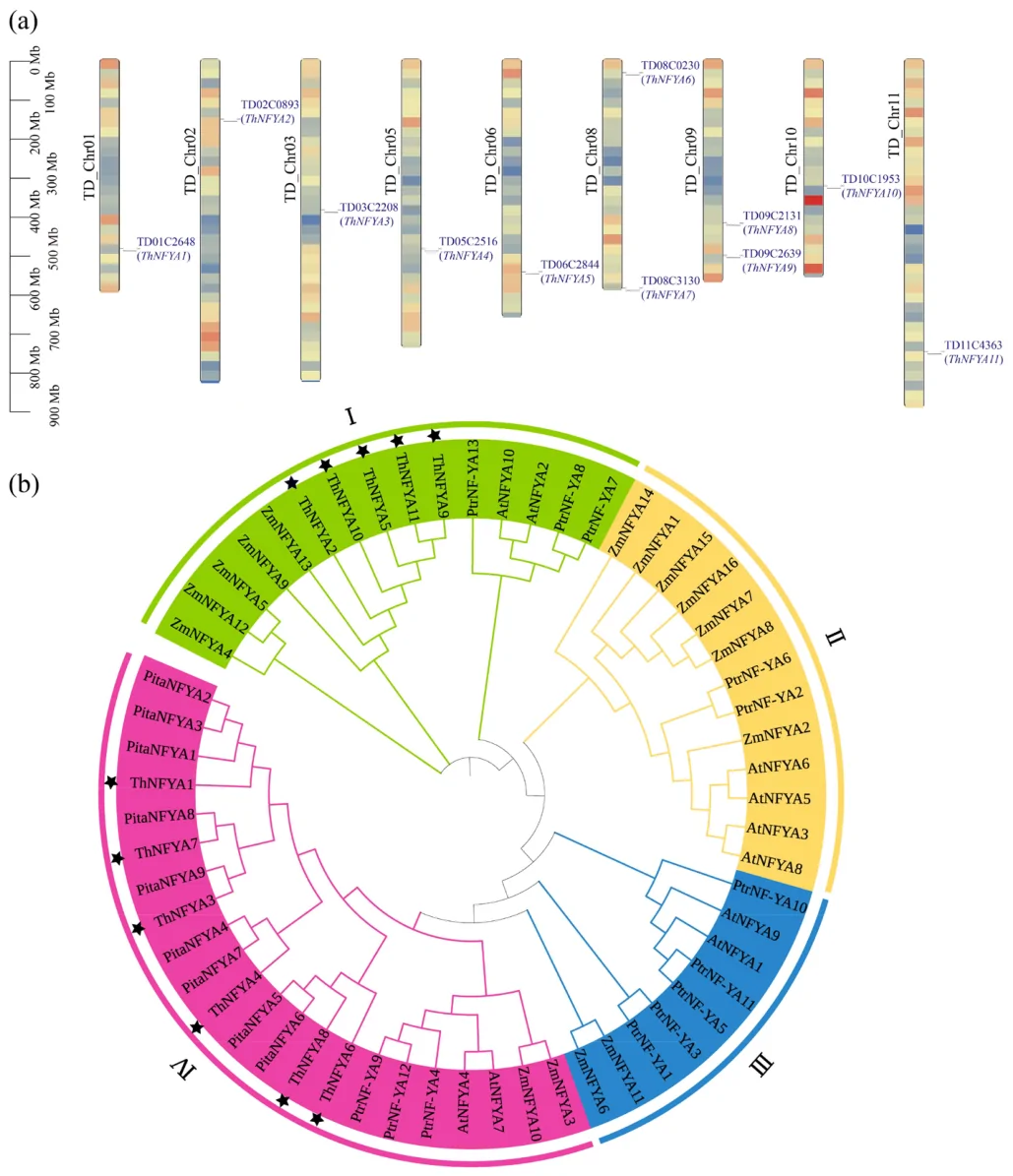
Chromosomal distribution (**a**) and phylogenetic analysis (**b**) of *ThNFYAs* in *Taxodium* hybrid ‘Zhongshanshan’. In panel a, the chromosomes are colored according to gene density, with red and blue assigned to high and low densities, respectively. In panel b, the phylogenetic tree includes *NFYAs* from *Arabidopsis thaliana* (At), *Pinus tabuliformis* (Pita), *Populus trichocarpa* (Ptr), and *Zea mays* (Zm).

A total of 11 putative *ThNFYA* genes were identified and designated as *ThNFYA1* to *ThNFYA11* based on their chromosomal positions (Figure 1a and Figure S1). These genes were distributed across chromosomes 1, 2, 3, 5, 6, 8, 9, 10 and 11 (Figure 1a). Each ThNFYA protein was confirmed to contain a complete CBF_NFYA domain (Pfam: PF02045) (Figure S2).

The physicochemical properties of the ThNFYAs were highly variable (Table S2). The amino acid lengths of ThNFYAs varied from 67 to 372 amino acids, with corresponding molecular weights ranging from 16.84 to 40.12 kDa (Table S2). Their isoelectric points (pI) values ranged from 6.13 to 10.24 (Table S2). Subcellular localization prediction showed that most ThNFYA proteins are nuclear localized (Table S2). However, ThNFYA1 and ThNFYA11 were predicted to localize in the cytoplasm and chloroplast, respectively (Table S2).

### 3.2. Phylogenetic Analysis of NFYA Family

To investigate the evolutionary relationships of NFYA in plants, the protein sequences of 59 members identified in *T*. hybrid ‘Zhongshanshan’ (Th), *Arabidopsis thaliana* (At), *Populus trichocarpa* (Ptr), *Pinus tabuliformis* (Pita) and *Zea mays* (Zm) were aligned to construct a phylogenetic tree (Figure 1b). The Phylogenetic analysis revealed that these NFYAs were divided into four categories (I–IV) (Figure 1b), which is consistent with a previous study [3]. All ThNFYAs were distributed exclusively in Classes I and IV. Specifically, five members (ThNFYA2, ThNFYA5, ThNFYA9, ThNFYA10 and ThNFYA11) were classified into Class I, and the remaining six (ThNFYA1, ThNFYA3, ThNFYA4, ThNFYA6, ThNFYA7 and ThNFYA8) were grouped into Class IV (Figure 1b). Notably, the class I category also contained AtNFYA2 and AtNFYA10, and the class IV clade included AtNFYA4 and AtNFYA7 (Figure 1b), implying that these ThNFYA members share close evolutionary relationships with their corresponding *Arabidopsis* homologs.

### 3.3. Gene Structure and Motif Characterization of ThNFYAs

**Figure 2:**
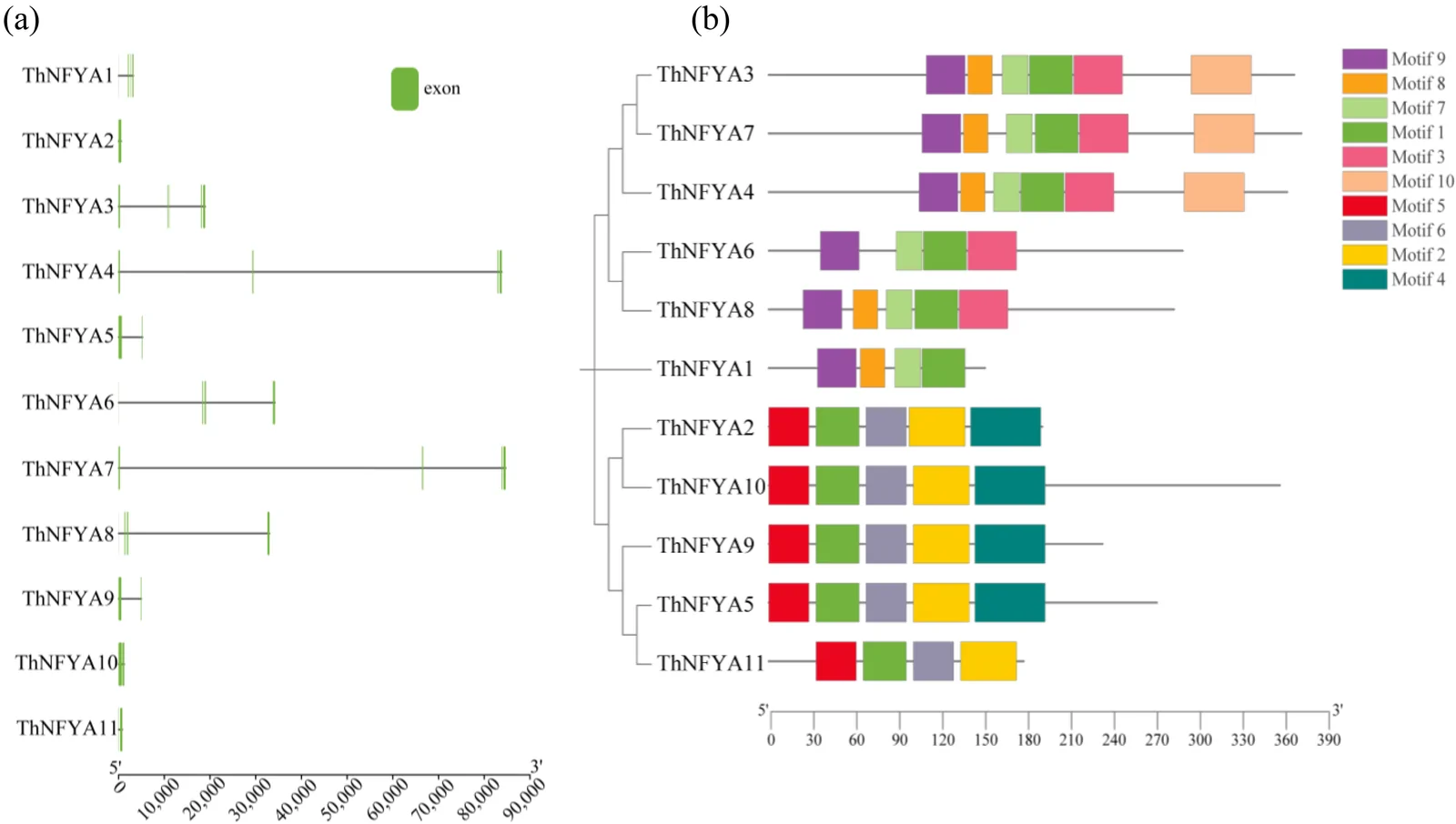
Gene structure (**a**) and conserved motifs (**b**) of *ThNFYA* family in *T*. hybrid ‘Zhongshanshan’. In panel a, introns are shown in black lines.

Because structure diversity within gene families is often associated with evolutionary dynamics, the exon-intron structures of *ThNFYAs* were analyzed. The exon number of *ThNFYAs* ranged from 1 to 5 (Figure 2a). *ThNFYA2* contained only one exon (visualized as an uninterrupted box without intron connectors in Figure 2a, while *ThNFYA3*, *ThNFYA4*, *ThNFYA7* and *ThNFYA8* possessed five exons (Figure 2a). To further investigate the conservation and diversity of *ThNFYAs*, the conserved motifs were identified. In total, 10 distinct motifs were predicted (Figure 2b). All ThNFYA proteins contained Motif 1 (Figure 2b). Intriguingly, motifs 2, 4, 5 and 6 were exclusively present in Class I *ThNFYAs*, while motifs 3, 7, 8, 9 and 10 were only existed in Class IV *ThNFYAs* (Figure 2b).

### 3.4. Cis-Acting Elements of ThNFYAs

**Figure 3:**
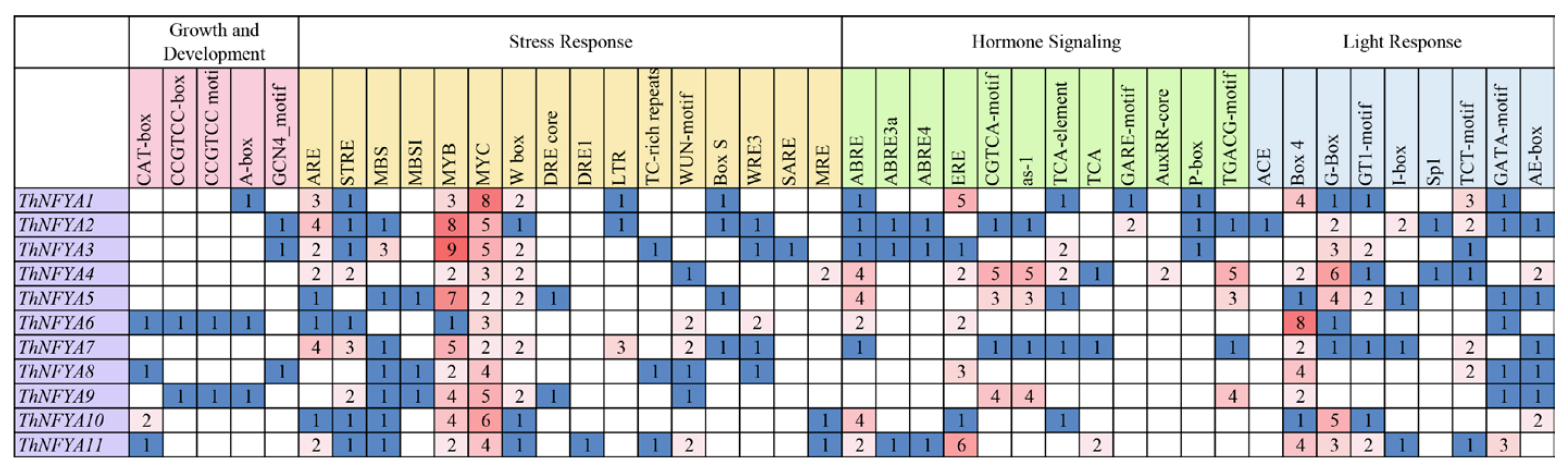
Cis-acting elements in the promoters of *ThNFYA* genes. The number and distribution of each element are shown for each *ThNFYA* gene.

To explore the putative functions of *ThNFYAs*, the promoters of the 11 *ThNFYAs* were retrieved and analyzed for putative cis-acting elements. *ThNFYA* promoters contained several types of cis-regulatory elements associated with growth and development, light responsiveness, stress responses and hormone signaling (Figure 3). Among them, stress-responsive elements were the most abundant across the *NFYA* family, with MYB, MYC, anaerobic response element (ARE), and W-box accounting for the highest proportion (Figure 3). Notably, MYB and MYC binding sites were found in the promoter of each *ThNFYA* (Figure 3). For hormone signaling elements, the ABA-responsive element (ABRE) and the ethylene-responsive elements (ERE) were widely detected in most *ThNFYA* genes (Figure 3). In particular, *ThNFYA1*, *ThNFYA8* and *ThNFYA11* contained 5, 3 and 6 ERE elements, respectively (Figure 3). For growth and development elements, each *ThNFYA* gene had 1–4 elements, except for ThNFYA4/5/7, which lacked these elements entirely (Figure 3). The prevalence of these stress- and hormonal-related cis-elements suggests that *ThNFYAs* may play pivotal roles in cope with environmental stresses.

### 3.5. Tissue-Specific Expression Profiles of ThNFYAs

**Figure 4:**
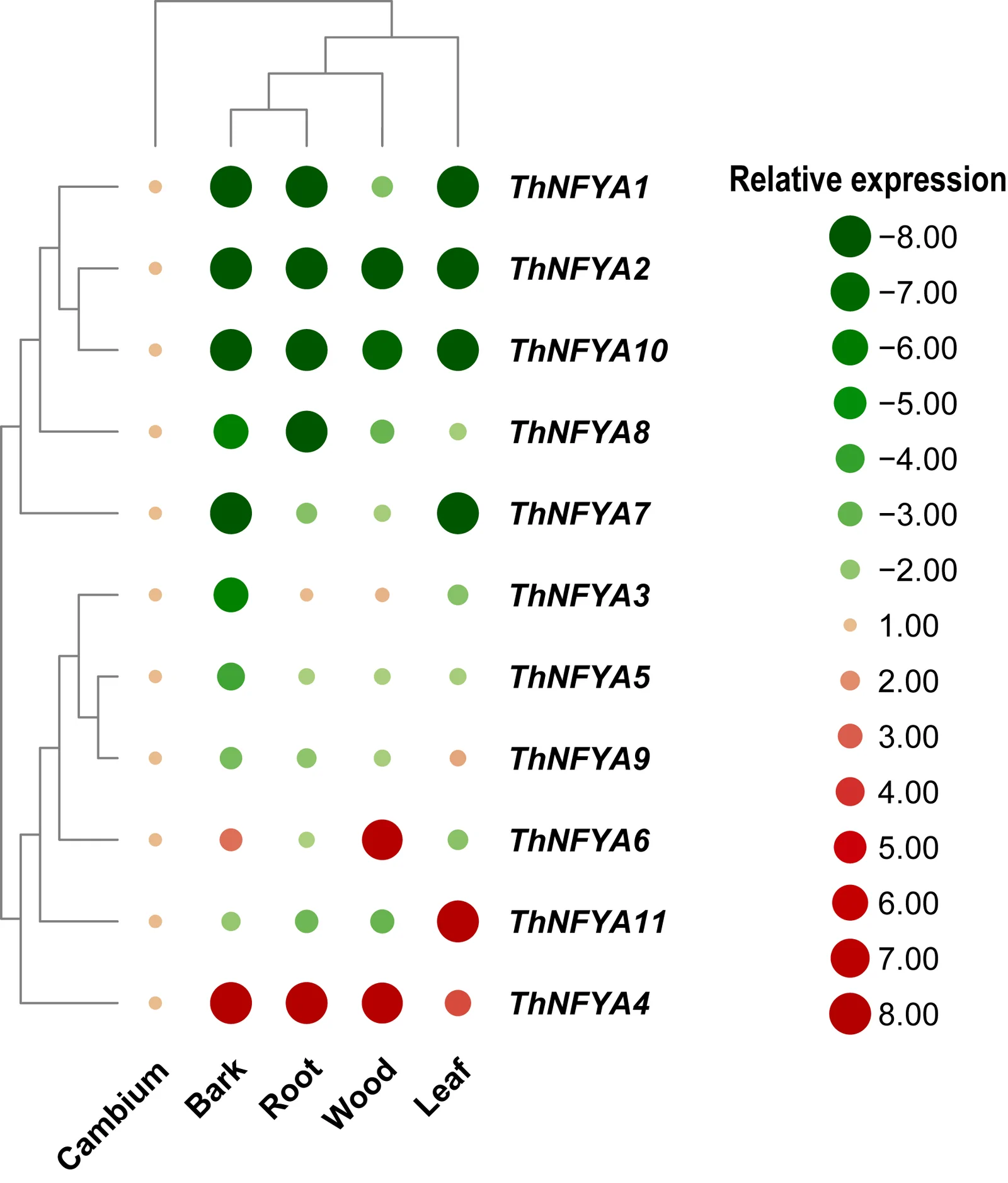
The expression levels of *ThNFYAs* in different tissues of *T*. hybrid ‘Zhongshanshan’.

The mRNA levels of *ThNFYAs* were examined in cambium, bark, wood, root and leaf tissues (Figure 4). *ThNFYA1*, *ThNFYA2*, *ThNFYA5*, *ThNFYA7*, *ThNFYA8* and *ThNFYA10* exhibited the highest transcript levels in the cambium and the lowest accumulation in the bark (Figure 4). *ThNFYA3* and *ThNFYA6* showed the highest mRNA levels in the wood and the lowest in the bark and leaves, respectively (Figure 4). *ThNFYA4* revealed higher mRNA levels in the roots, bark and wood and lower in other two tissues, whereas *ThNFYA9* exhibited the opposite pattern (Figure 4). *ThNFYA11* showed predominant expression in the leaves, while its transcript levels remained relatively low in other tissues (Figure 4).

### 3.6. Expression Patterns of ThNFYAs Responding to Salt and Drought Stresses

**Figure 5:**
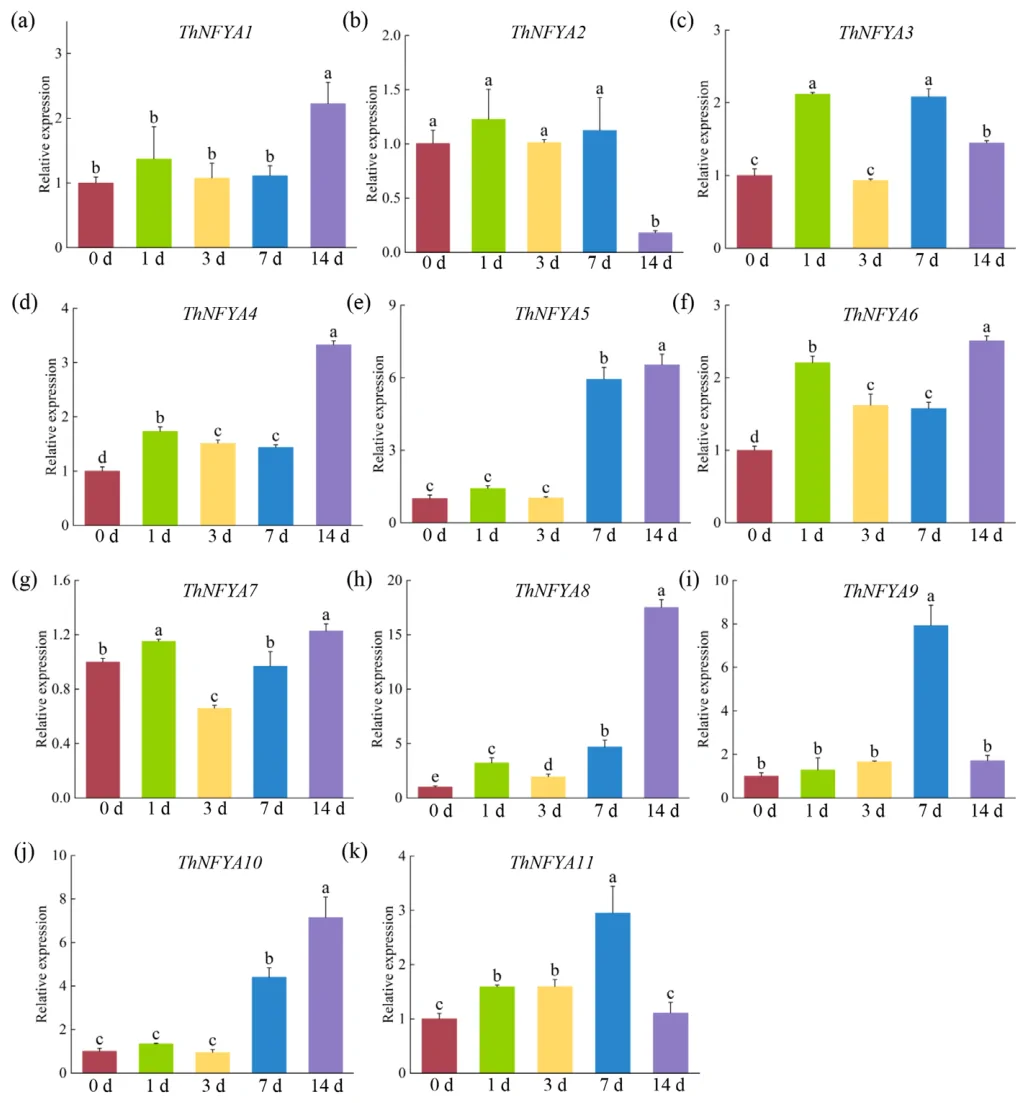
Gene expression levels of *ThNFYAs* in *T*. hybrid ‘Zhongshanshan’ roots treated with 200 mM NaCl for 0 (serving as control), 1, 3, 7, and 14 days, respectively. Bars indicate means ± SE (*n* = 3). Statistical analysis was performed using one-way ANOVA with Duncan’s multiple range test. Different letters on the bars indicate significant differences (*p* < 0.05) between the groups.

**Figure 6:**
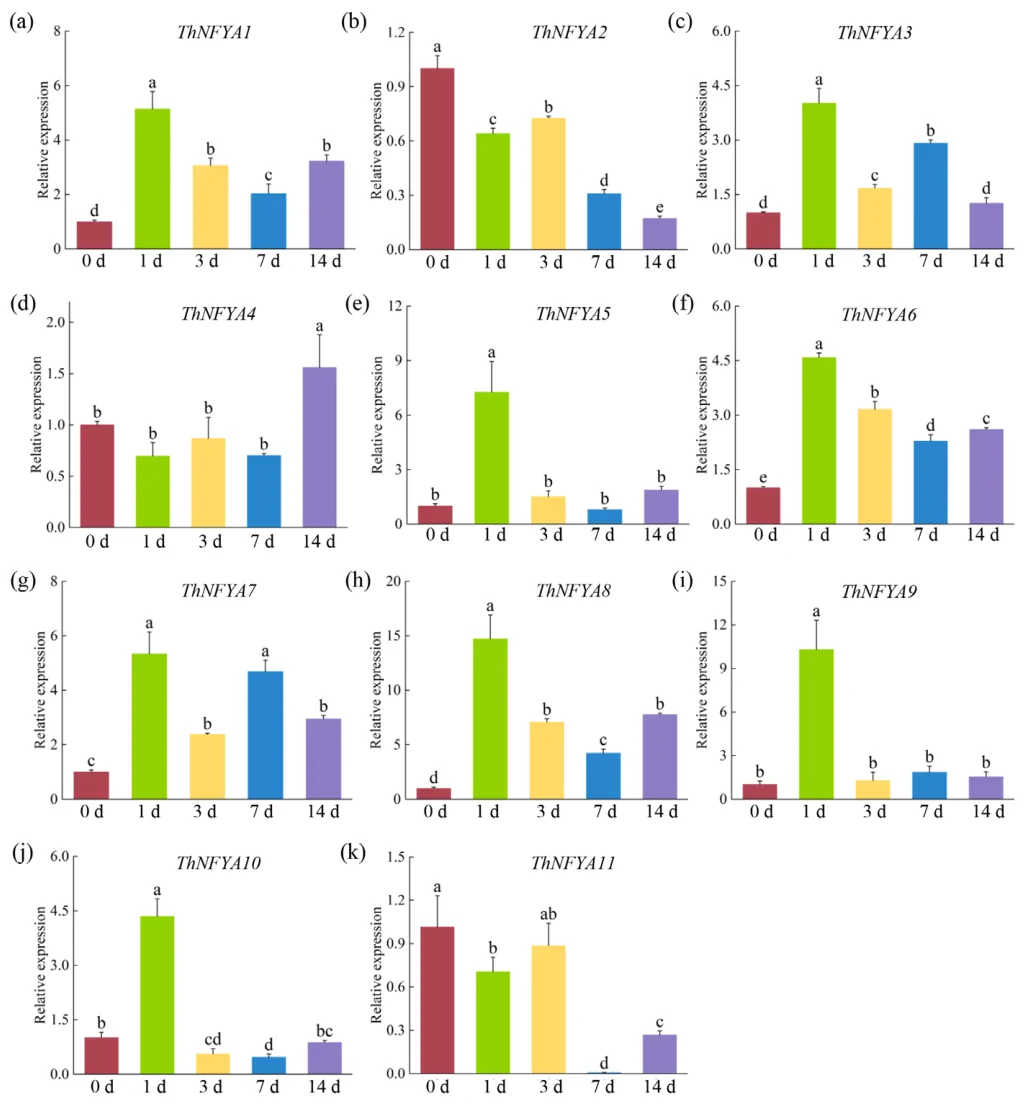
Gene expression levels of *ThNFYAs* in *T*. hybrid ‘Zhongshanshan’ leaves treated with 200 mM NaCl for 0 (serving as control), 1, 3, 7, and 14 days, respectively. Bars indicate means ± SE (*n* = 3). The information on statistical analysis is the same as mentioned in Figure 5.

**Figure 7:**
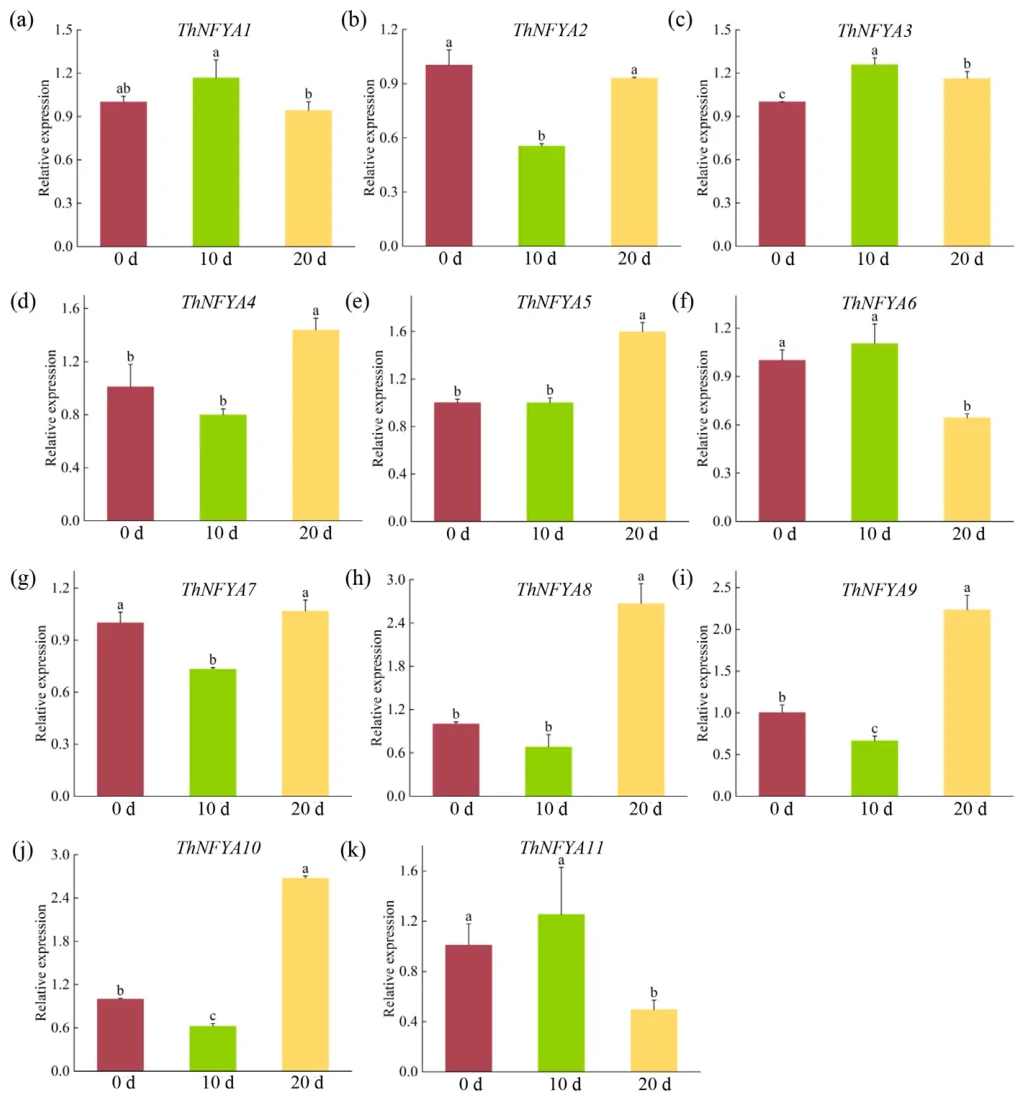
Gene expression levels of *ThNFYAs* in *T*. hybrid ‘Zhongshanshan’ roots subjected to water withholding for 0 (serving as control), 10 and 20 days, respectively. Bars indicate means ± SE (*n* = 3). The information on statistical analysis is the same as mentioned in Figure 5.

**Figure 8:**
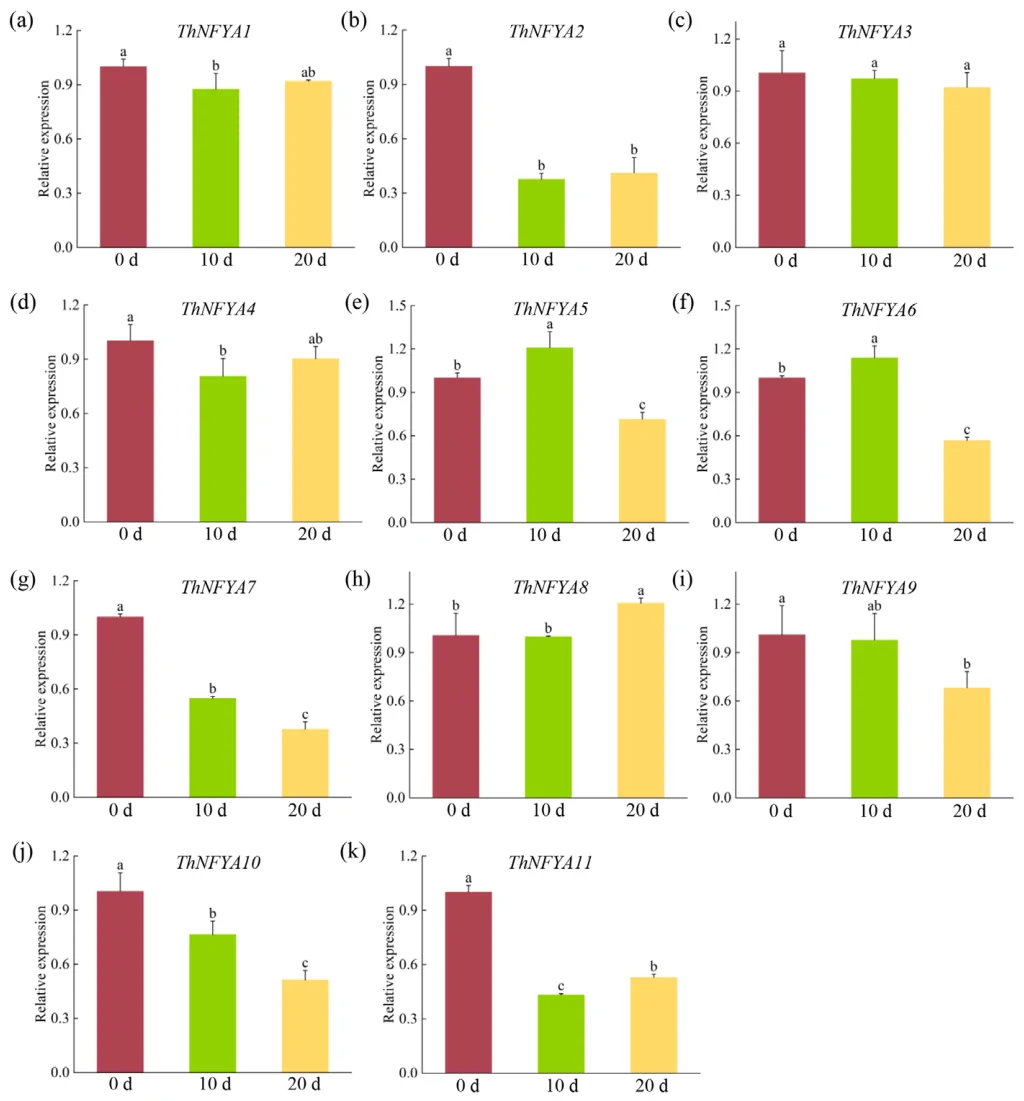
Gene expression levels of *ThNFYAs* in *T*. hybrid ‘Zhongshanshan’ leaves subjected to water withholding for 0 (serving as control), 10 and 20 days, respectively. Bars indicate means ± SE (*n* = 3). The information on statistical analysis is the same as mentioned in Figure 5.

**Figure 9:**
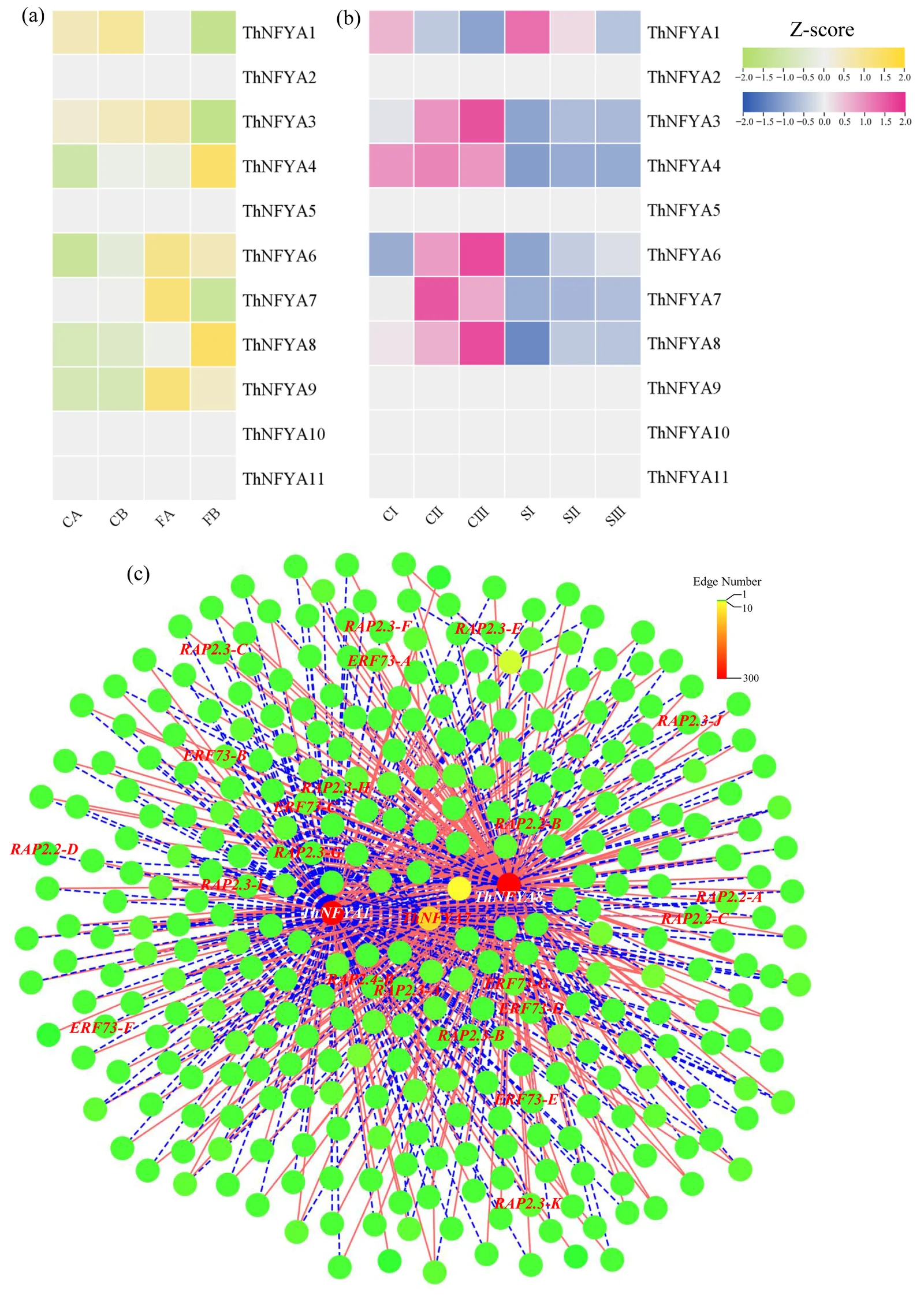
Gene expression profiles of *ThNFYAs* in the leaves (**a**) and wood (**b**), and co-expression network of *ThNFYAs* and other transcript factors in the leaves (**c**) of *T*. hybrid ‘Zhongshanshan’ in response to partial submergence. CA: leaves above flooding-water under control; CB: leaves below flooding-water under control; FA: leaves above flooding-water under partial submergence; FB: leaves below flooding-water under partial submergence. CI, CII and CIII represent the wood treated with control for one, two and three months, respectively. SI, SII and SIII represent the wood treated with partial submergence for one, two and three months, respectively. In panel (**c**), significant co-expression pairs (|r| > 0.7, *p* < 0.05) are shown. Red solid and blue dashed lines indicate positive and negative correlations, respectively.

Since stress-responsive cis-elements were abundantly identified in the promoter regions of *ThNFYAs*, their expression dynamics were assayed under salt, drought and flooding stresses (Figure 5, Figure 6, Figure 7, Figure 8 and Figure 9). Under salt stress, the mRNA levels of all *ThNFYAs* were increased in both roots and leaves, except for the downregulation of *ThNFYA2* in the roots and of *ThNFYA2* and *ThNFYA11* in the leaves (Figure 5 and Figure 6). In the roots, the majority of *ThNFYA* genes, including *ThNFYA1*, *ThNFYA4*-*ThNFYA8* and *ThNFYA10*, reached peak transcript levels at 14 days of exposure (Figure 5a,d–h,j), suggesting their involvement in long-term salt acclimation. *ThNFYA3* exhibited the highest mRNA levels at 1 and 7 days of salt stress, and *ThNFYA9* and *ThNFYA11* showed their peak transcript levels at 7 days of treatment (Figure 5c,i,k). In the leaves, most *ThNFYAs*, including *ThNFYA1*, *ThNFYA3* and *ThNFYA5*-*ThNFYA10*, reached their peak expression at day 1 of exposure (Figure 6a,c,e–j), reflecting a rapid systemic signaling role. *ThNFYA4* was upregulated at 14 days of salt stress (Figure 6d). Parallel physiological measurements under the same salt treatment revealed increased malondialdehyde (MDA) levels, along with elevated superoxide dismutase (SOD) and peroxidase (POD) activities in the roots of *T*. hybrid ‘Zhongshanshan’ [37].

Under drought stress, in the roots, the transcript level of *ThNFYA3* was increased, while those of *ThNFYA2*, *ThNFYA7*, *ThNFYA9* and *ThNFYA10* were decreased, and *ThNFYA1* and *ThNFYA8* remained unchanged at 10 days of drought (Figure 7a–c,g–j). Most *ThNFYAs*, including *ThNFYA3*-*ThNFYA5* and *ThNFYA8*-*ThNFYA10* were upregulated, while *ThNFYA6* and *ThNFYA11* were downregulated, and *ThNFYA1* was unaffected at 20 days of drought stress (Figure 7a,c–f,h–k). In the leaves, the transcript levels of *ThNFYA5* and *ThNFYA6* were stimulated, whereas those of *ThNFYA1*, *ThNFYA2*, *ThNFYA4*, *ThNFYA7*, *ThNFYA10* and *ThNFYA11* were reduced, and *ThNFYA8* remained stable at 10 days of drought (Figure 8a,b,d–h,j,k). Contrary to the patterns in the roots, most *ThNFYAs* in the leaves, including *ThNFYA2*, *ThNFYA5*-*ThNFYA7* and *ThNFYA9*-*ThNFYA11* were downregulated at 20 days of drought stress, whereas *ThNFYA8* was upregulated and *ThNFYA1* remained unaffected (Figure 8a,b,e–h,i–k). Parallel physiological measurements after 5 and 8 days of drought revealed elevated MDA levels and induced activities of SOD, POD and catalase (CAT) in *T*. hybrid ‘Zhongshanshan’ [38]. These results suggest that *ThNFYAs* exhibit tissue-specific and time-dependent expression patterns under drought stress, with a general shift from early responses to sustained downregulation in the leaves, implicating their diverse regulatory roles in drought acclimation.

### 3.7. Expression Patterns of ThNFYAs Responding to Flooding Stress

Given the extreme flooding tolerance of *T*. hybrid ‘Zhongshanshan’ and the presence of ARE element in the promoters of *ThNFYA* members, the expression of *ThNFYAs* was analyzed in the leaves and wood under partial submergence (Figure 9). Notably, our earlier physiological analyses have revealed that partial submergence triggers a suite of adaptive responses in this hybrid, including lower chlorophyll concentration, higher O_2_^−^ and H_2_O_2_ levels, and enhanced activities of SOD, POD, and CAT in the leaves and roots [26,27]. The transcript levels of *ThNFYA7* and *ThNFYA9* were increased in the leaves above flooding-water surface under partial submergence (SA vs. CA, Figure 9a). The mRNA levels of *ThNFYA8* and *ThNFYA9* were increased, whereas *ThNFYA1* was downregulated in the leaves below flooding-water surface under partial submergence (SB vs. CB, Figure 9a). In the wood, *ThNFYA7* and *ThNFYA8* were downregulated under one month of partial submergence, and *ThNFYA3*, *ThNFYA4*, *ThNFYA6* and *ThNFYA7* were downexpressed under two and three months of partial submergence (Figure 9b). These results suggest that *ThNFYAs* are differentially regulated in a tissue- and submergence duration-dependent manner, which may contribute to the adaptive flooding tolerance of *T*. hybrid ‘Zhongshanshan’.

### 3.8. Co-Expression Network Analysis of ThNFYAs

To explore potential regulatory relationships between *ThNFYAs* and other TFs under flooding, a co-expression network was constructed based on transcriptome data from the leaves under partial submergence [25]. Pearson correlation coefficients were calculated between the expression levels of *ThNFYAs* and those of significantly differentially expressed TFs (Tables S3–S5). In the co-expression network, six *ThNFYAs* (ThNFYA1/3/4/6/7/8) together with 330 TFs shared 722 interactions (Figure 9c). Noticeably, *ThNFYA1* emerged as the most prominent hub in this network (Figure 9c). It interacted with 319 TFs, and most interactions presented positive correlations (Figure 9c, Table S4). *ThNFYA8* and *ThNFYA7* ranked second and third, with 316 and 62 connections, respectively (Figure 9c, Table S4).

The co-expressed TFs included several well-characterized stress-responsive families (Figure 9c, Table S4). Among them, the *ERF* family was the most abundant, comprising 79 members, such as *ERF73* and *RAP2.2/2.3/2.4* (Figure 9c, Table S4). MYB (46 members) and WRKY (22 members) families also exhibited extensive connections with *ThNFYA1* and *ThNFYA8* (Figure 9c, Table S4). Members of these families are well documented to integrate multiple stress signals and regulate the expression of genes involved in ROS scavenging, osmotic adjustment and stomatal closure [39,40]. These results suggest that *ThNFYA1* and *ThNFYA8* may coordinate a broad array of stress-responsive TFs to contribute to flooding tolerance in *T*. hybrid ‘Zhongshanshan’.

## 4. Discussion

The *NFYA* family has been identified in several plant species, such as *Arabidopsis* [41], sorghum [42], maize [6], tomato [43], potato [44], cotton [45] and poplar [30], where these members play pivotal roles in regulating development and abiotic stresses response. In the present study, 11 *ThNFYAs* were identified in *T*. hybrid ‘Zhongshanshan’. This gene number is comparable to that of *Arabidopsis* (10 members), *P*. × *canescens* (13 members) and *P. trichocarpa* (13 members) [30]. Due to the highly repetitive nature of the *T. distichum* genome, we were unable to perform reliable synteny and duplication analyses in this study. However, a recent evolutionary investigation covering 320 plant species revealed that dispersed duplication dominates *NFY* gene expansion in gymnosperms [8]. The expansion pattern of *ThNFYAs* appears to be broadly consistent with the general evolutionary trajectory of gymnosperm *NFY* genes. Phylogenetic analysis revealed that five *ThNFYA* members (*ThNFYA2*, *ThNFYA5*, and *ThNFYA9*-*ThNFYA11*) belonged to Class I, while the remaining six (*ThNFYA1*, *ThNFYA3*, *ThNFYA4*, *ThNFYA6*-*ThNFYA8*) were grouped into to Class IV. Consistently, most members of Class IV *ThNFYAs* (*ThNFYA3*, *ThNFYA4*, *ThNFYA7* and *ThNFYA8*) contained five exons. Additionally, motifs 2, 4, 5 and 6 were uniquely existed in Class I members, while motifs 3, 7, 8, 9 and 10 were only present in Class IV members. Such class-specific motif distributions not only reinforce the phylogenetic grouping but may also suggest the possibility of functional divergence between the two subfamilies. Importantly, it should be emphasized that all these functional inferences are currently derived from phylogenetic and expression profiling. Without direct experimental validation, such as gene overexpression or knockout assays, these hypothetical functions remain speculative and require cautious interpretation.

In this study, class I members of *ThNFYAs* were phylogenetically clustered with *AtNFYA2* and *AtNFYA10*, raising the possibility that these *NFYAs* potentially share similar biological functions. In *Arabidopsis*, *AtNFYA2* and *AtNFYA10* have been functionally characterized as regulators of leaf growth, flowering time and root growth and branching [10,46]. Intriguingly, all Class I *ThNFYAs* exhibited pronounced tissue-specific expression patterns. Specifically, *ThNFYA2*, *ThNFYA5* and *ThNFYA10* showed the highest transcript levels in the cambium and the lowest in the bark. As the cambium is the primary tissue for secondary growth, this expression pattern might indicate their possible neo-functionalization or divergent roles in secondary growth. The overlapping expression profiles also imply potential functional redundancy among these members in regulating cambium development. However, this inference is based exclusively on spatial expression data without any direct measurements of wood properties, and further experimental verification is therefore required. Additionally, the expression levels of these *ThNFYAs* were markedly altered under salt and drought stresses, which may imply that they may could potentially act as balanced regulators coordinating development and defense responses in *T*. hybrid ‘Zhongshanshan’. However, such coordination is speculated based on correlative expression data, and direct experimental evidence is still lacking.

Phylogenetically, the class IV members of *ThNFYAs* group together with *AtNFYA4* and *AtNFYA7*, suggesting potential functional conservation among these NFYAs. In Arabidopsis, *AtNFYA4* and *AtNFYA7* have been functionally characterized as key players in stress adaptation [24,47]. In this study, all Class IV *ThNFYAs* were significantly differentially expressed under salt and drought stresses. Notably, the mRNA levels of *ThNFYA3*, *ThNFYA4*, *ThNFYA6*-*ThNFYA8* were reduced in the wood under partial submergence, indicating their possible roles in regulating wood properties in response to flooding. However, this interpretation is based solely on transcriptional evidence, any inference regarding the regulation of wood properties remains speculative. Furthermore, their promoters were highly enriched in stress-related cis-elements, particularly MYB, MYC and ABRE motifs. These cis-elements are recognized as integrators of ABA-dependent and ABA-independent stress signaling pathways [48]. These results may suggest that class IV *ThNFYAs* play pivotal roles in coordinating stress responses and wood property regulation. However, as these conclusions are based solely on cis-element predictions and transcriptional changes, future genetic transformation studies are required to functionally corroborate these hypotheses.

To further dissect key *ThNFYA* members potentially regulating flooding tolerance, a co-expression network linking *ThNFYAs* to other TFs was constructed based on leaf transcriptome data under partial submergence. *ThNFYA1* and *ThNFYA8* occupied the most central position in the co-expression network. The *ERF* family, such as *ERF73* and *RAP2.2/2.3/2.4*, comprised the largest proportion of these co-expressed TFs. Consistently, five and three ethylene-responsive ERE cis-elements were present in the promoter region of *ThNFYA1* and *ThNFYA8*, respectively. *ERF73* has been functionally identified as a positive modulator of waterlogging tolerance in kiwifruit, where it activates downstream hypoxia-responsive genes [49]. Additionally, *ERF*-VII subgroup members such as *RAP2.2* and *RAP2.3* are well-known master regulators of plant flooding responses [50,51]. They directly activate *alcohol dehydrogenase* (*ADH*) and *lactate dehydrogenase* (*LDH*) to enhance hypoxia tolerance under flooding [50,51]. These results raise the possibility that *ThNFYA1* and *ThNFYA8* might play key roles in regulating flooding stress response in *T*. hybrid ‘Zhongshanshan’. However, this network model is purely predictive and derived from transcriptional correlations without perturbational experiments. Further experimental validation of *ThNFYA1* and *ThNFYA8*, particularly genetic transformation, is required to conclusively establish its function in flooding tolerance.

## 5. Conclusions

Taken together, 11 *ThNFYA* genes were identified in *T*. hybrid ‘Zhongshanshan’, which were classified as two groups (Classes I and IV). These genes exhibited diverse physicochemical properties and showed tissue-specific expression patterns, with six members showing the highest transcript levels in the cambial tissues. Promoter cis-element analysis and expression profiling revealed that most *ThNFYAs* were responsive to salt, drought and flooding stresses, suggesting their potential involvement in abiotic stress adaptation. Intriguingly, the mRNA levels of class IV *ThNFYAs*, including *ThNFYA3*, *ThNFYA4*, *ThNFYA6*-*ThNFYA8*, were downregulated in the wood under partial submergence, raising a hypothesis that these genes may participate in wood properties modulation during flooding. Co-expression network analysis identified *ThNFYA1* and *ThNFYA8* as the most hub genes in partial submergence-treated leaves. Overall, these results indicate that *ThNFYAs* might play key roles in regulating development and abiotic stress response in *T*. hybrid ‘Zhongshanshan’.

## Data Availability

Data will be made available on request.

## Supplementary Materials

The following supporting information can be downloaded at https://www.mdpi.com/article/10.3390/life16081358/s1, Figure S1: Photos of *T*. hybrid ‘Zhongshanshan’; Figure S2: Diagram of ThNFYAs showing the position of the CBFB_NFYA domain. Table S1: Primers used for qRT-PCR; Table S2: Physicochemical properties of ThNFYAs in *T*. hybrid ‘Zhongshanshan’; Table S3: Significantly differentially expressed transcription factors co-expressed with *ThNFYAs* in the leaves of *T*. hybrid ‘Zhongshanshan’ in reponse to partial submergence; Table S4: Pearson correlation coefficients for co‑expression pairs between *ThNFYAs* and other transcription factors in the leaves of *T*. hybrid ‘Zhongshanshan’ in reponse to partial submergence; Table S5: *p*-values for co-expression pairs between ThNFYAs and other transcription factors in the leaves of *T*. hybrid ‘Zhongshanshan’ in reponse to partial submergence.
