# Supplementary material for: Genome-Wide Identification of the NFYA Family and Its Expression in Response to Abiotic Stress in Taxodium Hybrid ‘Zhongshanshan’
Source: Life (Basel). 2026 Aug 19;16(8):1358. doi: 10.3390/life16081358 (PMC13514229; doi:10.3390/life16081358)
Supplement: Supplementary file 1 [file life-16-01358-s001.zip › Supplentary figures.pdf]

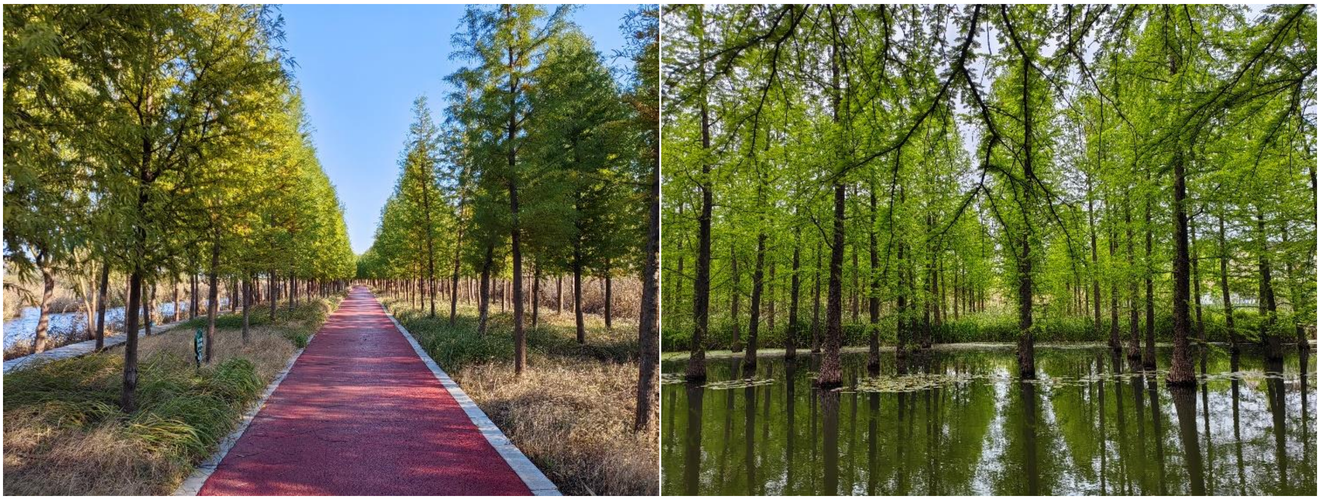

Figure S1 Photos of *T*. hybrid ‘Zhongshanshan’.

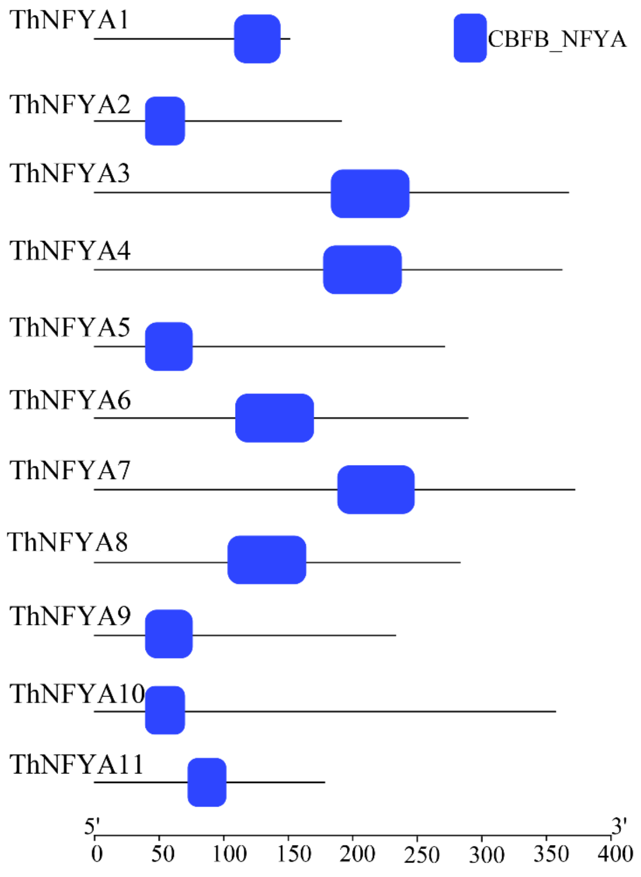

Figure S2 Diagram of ThNFYAs showing the position of the CBFB_NFYA domain.
